# Parkinson’s Disease–Mild Cognitive Impairment (PD-MCI): A Useful Summary of Update Knowledge

**DOI:** 10.3389/fnagi.2019.00303

**Published:** 2019-11-08

**Authors:** Davide Maria Cammisuli, Salvatore Massimiliano Cammisuli, Jonathan Fusi, Ferdinando Franzoni, Carlo Pruneti

**Affiliations:** ^1^Laboratories of Clinical Psychology, Clinical Psychophysiology and Clinical Neuropsychology, Department of Clinical and Experimental Medicine, University of Parma, Parma, Italy; ^2^School of Medicine, University of Pisa, Pisa, Italy; ^3^Department of Clinical and Experimental Medicine, University of Pisa, Pisa, Italy

**Keywords:** PD-MCI, epidemiology, pathophysiology, biomarkers, treatment

## Abstract

Mild cognitive impairment (MCI) is a common feature in Parkinson’s Disease (PD), even at the time of diagnosis. Some levels of heterogeneity in nature and severity of cognitive impairment and risk of conversion to Parkinson’s Disease Dementia (PDD) exist. This brief overview summarized the current understanding of MCI in PD, by considering the following major points: historical development of the clinical entity, evaluation, epidemiology, predictors and outcomes, neuroimaging findings, pathophysiology, treatment, and pharmacological and non-pharmacological intervention. MCI in PD represents a concept in evolution and plays a pivotal role in advancing our understanding of the disease mechanisms, with the ultimate goal of building effective strategies to prevent conversion into PDD. Challenges for future research are also discussed.

## Introduction

From its first conceptualization, mild cognitive impairment (MCI) has been recognized as a transitional state between normal cognition and overt dementia, especially of Alzheimer type (Petersen et al., 1999). Then, the Recommendations from the National Institute on Aging-Alzheimer’s Association workgroup pointed out the symptomatic pre-dementia phase of Alzheimer’s Disease (AD; i.e., MCI due to AD) by depicting a set of criteria with increasing levels of certainty, depending on the presence and nature of biomarker findings beyond cognitive impairment (Albert et al., 2011). More recently, the classification of the DSM 5 recognized the clinical entity of Minor Neurocognitive Disorder (NCD) for different neurodegenerative disorders including Parkinson’s Disease (PD), in order to establish a primary level of cognitive impairment in comparison with Major NCD, reporting a more severe deterioration (Regier et al., 2013).

In the last years, there has been an increasing emphasis on the identification of MCI in PD (PD-MCI). Interestingly, PD conception itself has progressively changed over time, moving from a “motor disease” to a “complex brain disease.” This turnover was supported by the presence of well-documented non-motor disorders, particularly cognitive deficits.

In order to provide clinicians and researchers a global perspective on the construct of MCI in PD, our review represents a brief summary of update knowledge on PD-MCI encompassing the following major points: historical development of the clinical entity, evaluation, epidemiology, predictors and outcomes, neuroimaging findings, pathophysiology, treatment, and pharmacological and non-pharmacological intervention.

## PD-MCI Evaluation

The heterogeneous evaluation to ascertain PD-MCI has led to substantial variation in the percentage of patients classified. To address this issue, the Movement Disorder Society (MDS) proposed standardized diagnostic criteria for PD-MCI (Litvan et al., 2012; Geurtsen et al., 2014). First, the presence of MCI in an established PD—diagnosed based on UK PD Brain Bank Criteria—reflecting a gradual decline as reported by either the patient or informant, not sufficient to significantly interfere with patients’ functional independance. Second, cognitive deficits are reported on a formal neuropsychological examination as performances approximately 1–2 standard deviations below appropriate norms. MDS diagnostic criteria encompassed two operationalization levels for neuropsychological examination. Level I criteria are based on impairment in a global cognitive test validated for use in PD or in a brief neuropsychological assessment (i.e., less than two tests for each of cognitive domains assessed, i.e., attention/working memory, executive function, memory, visuospatial skills, and language). Level II criteria, based on a comprehensive neuropsychological evaluation, require an impairment on at least two neuropsychological tests, represented by either two impaired tests in one cognitive domain or one impaired test in two different cognitive domains. The Level II criteria allow MCI subtyping (i.e., single domain or multiple domain).

Since 2012, an MDS Study Group was created for the validation of MCI in PD with the ultimate goal of comparing the two assessment levels in predicting PD-MCI conversion into Parkinson’s Disease Dementia (PDD). The study of Goldman et al. (2013) documented that MDS Task Force Level II recommendations provide a suitable framework for creating an efficient neuropsychological battery able to detect MCI in PD. This finding was then corroborated by other investigations (Stefanova et al., 2015; Bezdicek et al., 2017) while the utility of MDS Level I diagnostic criteria for MCI has been under debate for a long period (Szeto et al., 2015; Uysal-Cantürk et al., 2018). Lastly, the *MDS Study group* (Hoogland et al., 2019) concluded that Level I PD-MCI criteria classification also confers an independent contribution to the hazard of PDD while taking age, sex, education, motor sign severity, and depression into account, supporting its feasibility for neuropsychological diagnosis. With MDS Level II criteria, multiple domain impairment was more frequent than single-domain impairment, with predominant executive functioning, memory, and visuospatial deficits (Litvan et al., 2012; Cammisuli and Crowe, 2018). Furthermore, PD–Cognitive Rating Scale and Mattis Dementia Rating Scale-2 have been identified as suitable tests for distinguishing PD-MCI patients from cognitively normal PD patients (Koevoets et al., 2018).

Until now, biomarkers are not part of PD-MCI definition (Litvan et al., 2012). However, low levels of amyloid-β 42 (Aβ42) in cerebrospinal fluid (CFS) are associated with increased risk to develop cognitive impairment in PD (Alves et al., 2014). Most recently, a study observed a decreased network involving alpha activity over the occipital lobe, and increased network involving beta activity over the frontal lobe associated with a reduction over the parietal lobe, an increased network involving theta and delta activity over the frontal lobe, and a reduction of networks involving theta and delta activity in the parietal lobe. Furthermore, quantitative electroencephalography (EEG) analysis showed a significant decrease of alpha power spectral density (PSD) over the occipital regions and an increase of delta PSD over the left temporal region in PD-MCI as compared to patients with normal cognition (Mostile et al., 2019). Moreover, the correlation between PD-MCI and Rapid Eye Movement Sleep Behavior Disorder (Zhang et al., 2016) or olfactory dysfunction (Kawasaki et al., 2016) is widely confirmed. The relation between cognitive impairment and the presence of tau proteins in PD-MCI is currently under debate. Some studies suggest no association between them whereas high levels of T-tau and P-tau were shown to be associated with cognitive impairment in PD patients (Yu et al., 2014).

## Epidemiology, Predictors, and Outcome

Approximately 30%–40% of PD patients show cognitive impairment (Wojtala et al., 2019). Prevalence of PD-MCI may be an artifact of application methods of the criteria used for diagnosis (e.g., cut-offs of neuropsychological test scores, assessment levels, clinical settings, etc.). In the article depicting PD-MCI diagnostic criteria and assessment levels (Litvan et al., 2012), the mean prevalence of such a category was estimated at 27%. By considering cut-off scores of neurocognitive testing, Yarnall et al. (2014) reported a variable mean prevalence of PD-MCI of 65.8% (at 1 standard deviation), 42.5% (at 1.5 standard deviation) and 22.4% (at 2 standard deviation) below the normative values. Then, two cross-sectional studies estimated the prevalence of PD-MCI at 33% and 64%, respectively (Marras et al., 2013; Lawrence et al., 2016). In the Parkinson’s Disease Cognitive impairment Study (PACOS; Baschi et al., 2018; Monastero et al., 2018), including 659 non-demented PD patients, the prevalence of PD-MCI was 39.6% in the whole sample. Few longitudinal studies have assessed the incidence of PD-MCI according to the MDS Level II criteria (Broeders et al., 2013; Domellöf et al., 2015; Santangelo et al., 2015; Cholerton et al., 2018). The most recent study by Nicoletti et al. (2019) reported an incidence rate of PD-MCI of 184.0/1000 Pyar.

PD-MCI is associated with increasing age, male gender, and lower level of education, and its development seems to be influenced by a number of non-motor features, including sleep behavior disorders, severity of daytime sleepiness, and autonomic impairment as well as depression and anxiety (Palavra et al., 2013). Motor disease severity, akinetic rigid phenotype (Wojtala et al., 2019), and comorbidity with metabolic syndrome (Peng et al., 2018) seem to be associated with cognitive deterioration in PD, too. Conversely, higher levels of physical exercise, including strength, aerobic, and balance training in midlife, are associated with a lower risk of PD, and patients who remain physically active report greater quality of life and lower rates of falls and fractures (Mantri et al., 2018). By contrast, physical inactivity is a contributing factor in many diseases, including metabolic syndrome, characterized by inflammation and oxidative stress, thought also as presumed pathogenetic mechanisms of PD (LaHue et al., 2016).

Although it is difficult to compare among studies, a considerable amount of PD-MCI patients progress to PDD (19%–62%) when they are followed from 2 to 5 years after diagnosis (Wood et al., 2016). Recently, conversion rates from PD-MCI to PDD have been reported by one study more precisely as from 39% to 50% at a 5-years follow-up (Pedersen et al., 2017). Moreover, findings from longitudinal studies also documented significant rates of reversion to normal cognition of PD-MCI patients ranging from 11% to 27.8% at the same follow-up period (Domellöf et al., 2015; Weil et al., 2018).

## Neuroimaging Findings and Pathophysiology

The pattern of PD-MCI is characterized by cortical atrophy, mainly involving the right anterior temporal, left prefrontal and insular, and right parietal and occipital areas (Melzer et al., 2013). Atrophy proceeds to subcortical areas as well in the course of the pathology (Melzer et al., 2013). Diffusion tensor imaging (DTI) and diffusion-weighted imaging magnetic resonance imaging (MRI) techniques were used to investigate such brain regions. A recent study (Xiuqin et al., 2018) aiming at identifying the specific neuroanatomical alterations in early drug-naive PD-MCI patients by using voxel-based morphometry (VBM) showed that they exhibit atrophy in the right entorhinal cortex (ENT) in comparison to PD patients with normal cognition. A resting-state functional MRI study (Wang et al., 2018) documented hyperactivity—reflecting a compensatory mechanism—in the opercular part of right inferior frontal gyrus and hypoactivity—associated with cognitive decline—in the occipital areas in early PD with MCI. Studies carried out by positron emission tomography with f18-fluorodeoxyglucose (FDG-PET) showed a reduced metabolism of posterior brain cortical areas of PD-MCI patients. An investigation using the single-photon emission computed tomography imaging of dopamine transporters (DAT-SPECT) revealed that dopamine uptake reduction on caudate nucleus may better predict cognitive decline in PD when this technique is associated to other variables, such as patients’ age and CSF biomarkers (Schrag et al., 2017). A very recent study with a multi delay multiparametric arterial spin labeling investigated cerebral perfusion including cerebral blood flow (CBF) and arterial transit time (ATT) in PD-MCI patients using a voxel-based brain analysis and showed that ATT may be a more sensitive marker than CBF, by highlighting the potential role of thalamus and inferior parietal region in detecting MCI on early stage of PD (Suo et al., 2019).

Moreover, some studies suggest heterogeneous underlying neuropathology characterized by the presence of Lewy bodies in neocortical and/or limbic areas of PD-MCI patients (Jellinger, 2010). Recent studies show that white matter abnormalities, as revealed by DTI, precede for gray matter atrophy in non-demented PD, although the role of such degeneration in white matter in cognitive decline in PD is still debated (Mak et al., 2015).

Finally, there is increasing evidence linking small vessel disease (SVD) to motor and cognitive symptoms in PD. SVD, including white matter hyperintensities, lacunes, perivascular spaces, and microbleeds, is associated to functional and cognitive decline in PD patients (Foo and Kandiah, 2016). However, the lack of uniform criteria and standardized imaging techniques currently hamper research progress exploring the relationship between cerebrovascular disease and PD.

## Pharmacological and Non-Pharmacological Treatment

Currently, there are no effective drugs for improving cognition in PD-MCI. A recent study has hypothesized that treatment with atomoxetine would improve executive functioning in patients with PD-MCI, including executive control, set-shifting, and working memory (Hinson et al., 2017). Cholinergic degeneration could contribute to gait impairments, cognitive impairment, psychosis, and REM-sleep disturbances, and rivastigmine may be particularly useful for cognitive dysfunction also in PD patients (Perez-Lloret et al., 2016). Novel trials are nowadays implemented to test inhibitor of non-receptor tyrosine kinase Abelson (c-Abl kinase) activity, among which nilotinib seems to have success (phase 1 clinical trial on animal models; Lindholm et al., 2016). Moreover, prasinezumab (PRX002)—an anti-α-synuclein monoclonal antibody—is being used as a first-in-human phase 1 clinical trial (Schenk et al., 2017). A very recent investigation has shown that the use of cholinesterase inhibitors or memantine—a blocking of N-Methyl-D-aspartate (NMDA) receptors—may play a role in improving cognitive and motor function as well as behavioral symptoms in PD patients (Meng et al., 2019). Furthermore, the gut micriobiome claimed as an etiopathogenetic factor would suggest a novel target in PD pharmacotherapy (Mulroy and Bhatia, 2019).

The risk of developing PD appeared to be inversely associated with physical activity (PA) practiced during life (Sutoo and Akiyama, 2003; Chen et al., 2005; Xu et al., 2010; Alonso-Frech et al., 2011). Moderate-to-vigorous exercise in early adult life was associated with a highly significant 60% lower PD risk in men and a lower risk reduction in women (Chen et al., 2005; Ahlskog, 2011). In addition, the major positive adaptation of PA is related to increased insulin/IGF sensitivity and ketone utilization; increased expression of BDNF, FGF2, and VEGF with improved bioenergetics; and enhanced neuroplasticity (Mattson, 2014). It has been demonstrated that aerobic exercise, stretching, and balance training improve motor functions in PD patients (States et al., 2011). In particular, PA enables PD patients to maintain their psychomotor learning abilities (Paillard et al., 2015). The Parkinson Progression Markers Initiative (PPMI) documented that only 47% (in a total of 383 PD patients) reported activity consistent with the American Heart Association (AHA) Recommendation (i.e., 150 min of moderate or 75 min of vigorous PA weekly performed; Marek et al., 2018). PA, such as dance and cognitive exercise associated with motor training, has been shown to be effective in improving global cognitive function, processing speed, sustained attention, and mental flexibility in PD patients of mild/moderate stage, with a 6-years clinical diagnosis of disease (da Silva et al., 2018). Specifically, treadmill training performed three times a week for about 60 min in a total of 24 weeks produced a larger improvement in cognition (da Silva et al., 2018). PD patients performing progressive resistance training improved cardiac sympathetic modulation, as measured by heart rate variability and blood pressure response, too (Kanegusuku et al., 2017).

Available knowledge on the mechanisms involved in the protective role of PA for PD patients resulted from animal models. In particular, PA reduces the alteration of the dopaminergic neurons in the substantia nigra and contributes towards reconstituting the basal ganglia functions involved in motor commands (Speelman et al., 2011). Such action is related to an increased concentration of brain-derived neurotrophic factor (Wu et al., 2011). Aerobic exercise for PD rats on sessions lasting from 20 to 60 min, performed 5 days a week for 4 weeks, can restore the expression of the glial fibrillary acidic protein in the dorsal striatum (Dutra et al., 2012). Furthermore, regular and continuous aerobic training of rats over a period of 18 months also had a neuroprotective effect on the cerebellum (Larsen et al., 2000), which is centrally involved in movement and balance control.

Evidences from literature have further supported the potential role played by cognitive training (CT) and transcranial stimulation as complementary strategies to pharmacological treatment in PD-MCI. A randomized controlled trial including PD-MCI patients (Petrelli et al., 2014) showed specific effects of cognitive multicomponent structured (i.e., NeuroVitalis) and non-structured (i.e., “Mentally Fit”) interventions on short-term and working memory and on depression, respectively. Repeated sessions of anodal transcranial direct current stimulation (tDCS) on PD-MCI patients lead to a reduction of depressive symptoms and to increased performance in motor abilities and cognitive functions (especially in global cognition and verbal fluency), with stable effects at 3-months follow-up (Manenti et al., 2016). Remarkably, it has been shown that tDCS over the medial prefrontal cortex enhances cognitive Theory of Mind ability, suggesting this technique as useful to improve the social cognition of PD-MCI patients, too (Adenzato et al., 2019). A pilot study using a composite strategy of computer-based CT (i.e., 30 min, 4 days a week for 4 weeks) and tDCS over dorsolateral prefrontal cortex administered during executive tasks showed that tDCS extend the effect of CT on learning tests (Biundo et al., 2015).

## Discussion

PD-MCI is a syndrome defined by clinical, cognitive, and functional criteria. PD patients may present with non-motor features that are associated with increased disability and reduced quality of life and their relation with dopaminergic treatment needs to be further explored (Wishart and Macphee, 2011). A significant heterogeneity exists with respect to cognitive profiles found in PD-MCI patients that are likely predictive of distinct outcomes regarding the occurrence of *PDD*, with single domain (*amnestic*) MCI patients at higher risk (Hoogland et al., 2018). Complete neuropsychological testing of Level II criteria represents the gold standard for the diagnosis of PD-MCI, even though additional research is needed to identify a neuropsychological test battery for multisite international protocols that is sensitive to cognitive decline and responsive to therapeutic interventions.

To date, the validity of MCI in PD as a clinical entity is sustained by converging neuropsychological data, whereas research on biomarkers should be improved, even if emerging evidences from neuroimaging studies in differentiating neuroanatomical features of PD-MCI patients from normal controls and PDD patients already exist. Other biomarkers can be investigated in plasma, too. Indeed, plasma α-synuclein levels are reported as significantly higher in PD patients with dementia than in PD patients with MCI or normal cognition, by suggesting that α-synuclein may assist clinicians in identifying progression towards degeneration (Lin et al., 2017). Moreover, also in the case of PD-MCI, it should be useful for prognostic and therapeutic purposes to approach a diagnostic algorithm orienting clinical judgment as that built for “traditional” MCI (Petersen et al., 2014).

Motor phenotypes and their neuropsychological patterns in PD-MCI patients (i.e., tremor-dominant, akinetic-rigid, and mixed profile) should be investigated longitudinally, in order to detect PD-MCI patients at higher risk, given that tremor-dominant phenotype tends to progress into akinetic-rigid phenotype in the course of PD progression (Wojtala et al., 2019).

Further research should improve therapeutic trials for PD-MCI examining the effects of pharmacological agents affecting neurotransmission to better investigate the correlation between α-synuclein and cognitive decline of PD-MCI patients, implement CT protocols by large randomized controlled trials with longitudinal follow-ups even in association with tDCS, and explore the role of combined physical exercise and CT interventions.

There is now an increasing body of clinical evidence suggesting that physical exercise is a beneficial, cost-effective, and low-risk intervention for patients with PD. According to Fayyaz et al. (2018), optimally prescribed physical exercise programs may modulate neurophysiological processes possibly slowing down PD symptoms, especially for patients with moderate cognitive and physical impairment. PA has been shown to attenuate dopaminergic neuron damage and reduce cellular inflammation and oxidative stress (Nardello et al., 2017), thus playing a pivotal role in non-pharmacological interventions. Regular and moderate PA induces positive modification on cortisol level, endothelium function, and reduced inflammation. Indeed, endothelium-dependent flow-mediated dilation has been observed to be lower in sedentary older subjects as compared with older athletes (Franzoni et al., 2005). PA is a good method even in pathological conditions, as shown by Donia et al. (2019), reporting that acute exercise can positively induce changes in inflammation with reduction of TNF-α.

## Author Contributions

DC and SC equally contributed as first authors and made the most substantial contribution in writing the manuscript. JF and FF took care of the part pertaining to physical exercise interventions. CP revised the manuscript for intellectual content.

## Conflict of Interest

The authors declare that the research was conducted in the absence of any commercial or financial relationships that could be construed as a potential conflict of interest.
